# Long-term impact of hymenoptera venom immunotherapy on clinical course, immune parameters, and psychosocial aspects 

**DOI:** 10.5414/ALX02175E

**Published:** 2021-01-26

**Authors:** Jan Adelmeyer, Julia Pickert, Wolfgang Pfützner, Christian Möbs

**Affiliations:** 1 Clinical and Experimental Allergology, Department of Dermatology and Allergology, Philipps-Universität Marburg, Marburg, and; 2 Department of Dermatology and Allergology, Allergy Center Hessen, University Medical Center Marburg, Marburg, Germany; *Authors contributed equally.

**Keywords:** hymenoptera venom, allergy, venom immunotherapy, tolerance, IgE, IgG, quality of life

## Abstract

Background: Venom immunotherapy (VIT) is highly efficient in subjects suffering from IgE-mediated allergy to hymenoptera venom (HV), and VIT results in substantial improvement of quality of life (QoL). However, VIT-induced tolerance may be lost over time after cessation of treatment, putting patients at risk of re-sting anaphylaxis. Materials and methods: To study the effect of VIT on maintenance of HV tolerance we evaluated the natural history of 54 patients who were treated with VIT up to 29 years ago, with a special focus on re-stings and their subsequent course. Furthermore, we analyzed HV-specific IgE, IgG, and IgG4 antibody titers. Finally, we assessed the long-term impact of VIT on various psychosocial aspects like dealing with hymenoptera exposures, daily life activities, self-assurance, and personal environment. Results: 29 (53.7%) subjects experienced at least one re-sting after stopping VIT, with 23 (79%) showing no systemic reaction (SR). Eleven of these (37.9%) took emergency drugs as a safety measurement. Six individuals (21%) showed loss of tolerance experiencing an anaphylactic reaction. No difference in HV-specific IgE, IgG4, or IgG antibody concentrations was noticed among the different patients. Subjects who tolerated a re-sting without applying emergency drugs felt least affected in their social-behavioral leisure activities when hymenoptera were around or by anxiety for new stings. Conclusion: VIT leads to long-term tolerance in the majority of HV-allergic patients, however, ~ 1/5 may lose protection over time, arguing for continued follow-up on VIT-treated subjects and keeping them equipped with an emergency kit. Notably, VIT also results in a lasting, strong impact on self-assurance and sense of well-being in individuals who tolerated a re-sting without employing emergency drugs, which emphasizes the need to use them only in case of systemic symptoms after stopping successful VIT.

## Introduction 

IgE-mediated reactions to hymenoptera venom (HV) affect ~ 3.5% of the German population [1]. Clinical pictures range from cutaneous symptoms (urticaria, angioedema) to severe anaphylaxis potentially resulting in cardiac arrest. Since the outcome of a bee or wasp sting is unpredictable, HV-allergic patients need to be comprehensively instructed as to how a sting can be avoided. Moreover, they always have to carry an emergency kit encompassing an adrenalin injector as well as oral corticosteroids and antihistamines [1]. 

The only causal treatment is venom-immunotherapy (VIT), which has been shown as highly efficient in providing HV tolerance [2]. Furthermore, VIT also results in improved quality of life (QoL) [3, 4, 5]. A recent systematic review of clinical trials revealed that only 2.7% of patients with either HV or ant venom allergy still experienced anaphylaxis when stung again after receiving VIT compared to 39.8% of non-treated subjects [6]. In general, HV tolerance is quickly established by VIT, and almost 90% of patients are already protected against a hymenoptera sting 1 week after reaching the maintenance dose [7]. However, it is not well known for how long allergen tolerance will last after VIT, generally applied for 3 – 5 years, is finished [8]. Thus, it is recommended that patients with certain risk factors like increased exposure to bees/wasps or enhanced probability of developing severe anaphylactic reactions because of mastocytosis or cardiac co-morbidities continue VIT as long as these risks are prevalent [8]. Moreover, patients are advised to permanently and meticulously execute measures of sting avoidance. 

However, avoidance of re-stings is difficult to assure, as has been demonstrated by a study on HV-allergic children, of which 43% have been stung again during the next 10 years following VIT [9]. Thus, there is a great need for enhanced knowledge about the long-term course of HV allergy after terminating VIT and potential influencing factors. While a number of studies has been conducted on this topic, many of them are hampered in their significance by rather short periods of observation, high variability in the treatment of the studied patients (like in terms of VIT duration or HV dosage), and different assessments of tolerance maintenance [9, 10, 11, 12, 13, 14, 15, 16, 17, 18, 19, 20, 21, 22, 23, 24, 25, 26, 27, 28, 29]. Of note, recent corresponding investigations for the German population are missing. We therefore performed a survey on adult HV patients formerly treated by VIT at the Department of Dermatology and Allergology of the University Medical Center Marburg, exploring the natural history of their allergy after finishing VIT, and compared these with the findings of the previous reports. In addition, in vitro analysis of HV-specific IgE, IgG, and IgG4 antibodies was performed to evaluate how these parameters might relate to long-lasting tolerance. Furthermore, we were interested in knowing about the long-term consequences of VIT on the behavior of HV-allergic patients during their professional and leisure activities as well as on individual personal aspects affecting their daily life. 

## Patients and methods 

### Patients 

Patients were recruited retrospectively from the data files of the Department of Dermatology and Allergology, Allergy Center Hessen at the University Medical Center Marburg. Inclusion criteria were an age above 17 years and former treatment for HV allergy by VIT with a minimum duration of 3 years (Table 1). Subjects were excluded from the study if VIT had not been completed at the time of evaluation or if they were not capable of consent to the study terms. The study was approved by the Ethics Committee of the Medical Faculty of the Philipps-Universität Marburg. 

### Analysis of serum antibodies 

Total IgE and HV-specific IgE, IgG, and IgG4 serum antibodies were determined by ImmunoCAP assay (Thermo Fisher Scientific, Freiburg, Germany) according to the manufacturer’s instructions. 

### Clinical survey and questionnaires 

Individual survey data comprised general clinical information, severity of the initial systemic reaction (SR) to a hymenoptera sting according to the criteria of Ring and Messmer [30], duration and course of VIT, and the time thereafter, especially in regard of sting challenges (SC) and/or field stings (FS) and their outcome, including implemented therapeutic measurements. Furthermore, patients were asked to judge six statements evaluating the long-term impact of VIT on various behavioral and personal aspects related to their HV allergy (Table 2). Answers were documented on a visual analogue scale (VAS) from 0 (defined as “*does not apply at all*”) to 10 (defined as “*applies fully and completely*”). In addition, overall QoL was evaluated by a standardized WHO questionnaire, the WHOQOL-BREF [31, 32]. This instrument for generic self-assessment of QoL consists of 24 questions in the four domains “*physical health”* (7 items), “*psychological health”* (6 items), “*social relationships”* (3 items), and “*environment”* (8 items) as well as two additional questions regarding the “*overall QoL”* (2 items) of the patients. 

## Results 

### Clinical course 

**Figure 1.**
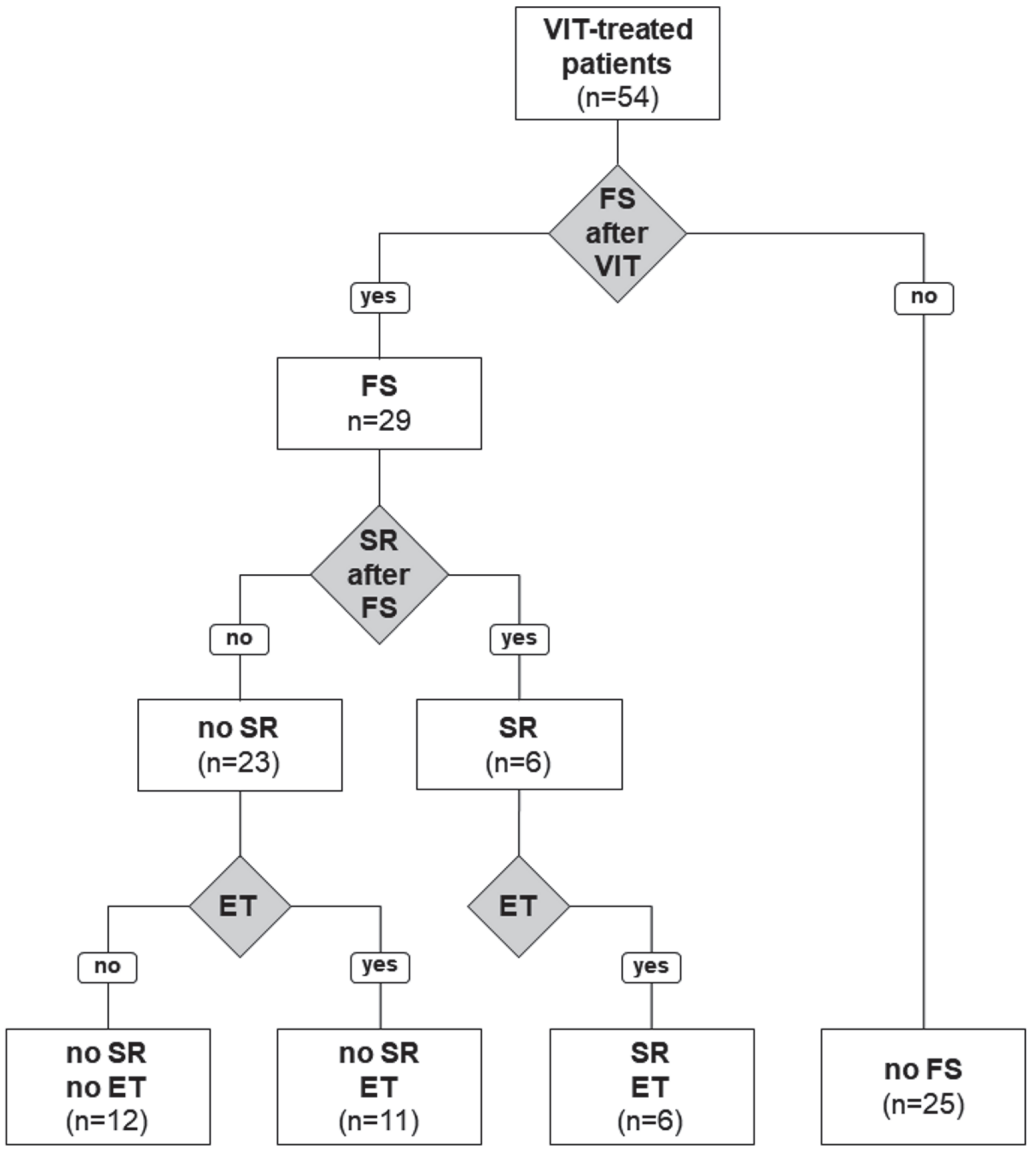
Distribution of patients treated with hymenoptera venom immunotherapy (VIT). ET = emergency treatment; FS = field sting; SR = systemic reaction.

A total of 54 patients were included in the study (Figure 1). Five of the 59 initially interviewed patients had experienced only a large local reaction and were thus excluded from further evaluation. The remaining 54 patients (28 females, 26 males; mean age 59 years) were treated by VIT for a median duration of 3 years (range 3 – 11 years) and interviewed 1 – 29 years (median 9 years) after treatment had been terminated (for clinical data see Table 1). 40 patients (74.1%) had received VIT with wasp venom, 10 (18.5%) with bee venom, and 4 (7.4%) were treated with both venoms. Personal history revealed a severity grade of the primary SR of grade I in 3 subjects (5.56%), grade II in 14 (25.93%), grade III in 30 (55.56%), and grade IV in 1 (1.85%) according to Ring and Messmer [30]. Six patients (11.11%) reporting systemic symptoms could not precisely specify their distinct nature due to the fairly distant event. 

29 of the total patients (53.7%) with former SR were stung again at least once with the HV allergy-eliciting insect after they had finished VIT (Table 1). Of these, 41% tolerated the FS without any therapeutic intervention (n = 12; 22.2% of all). Another 11 individuals (37.9% of re-stung patients; 20.4% of total) also did not develop an SR, but as a precaution took at least one of the drugs from their emergency treatment (ET) kit (usually consisting of an i.m. self-administrable adrenalin injector, an oral corticosteroid and antihistamine) or were treated by a consulted physician. The individual drugs and the time points of their application could not exactly be assessed due to the events dating far back. For this reason, it was difficult to estimate if patients were still HV-tolerant because of the former VIT or whether the ET might have prevented a potential SR. Nevertheless, 6 of the 29 re-stung subjects (20.7%, 11.1% of total) developed an SR, thus showing a loss of allergen tolerance despite taking emergency drugs or receiving medical treatment. 

### Immunological data 

**Hymenoptera venom-specific IgE, IgG, and IgG4 antibodies**

**Figure 2.**
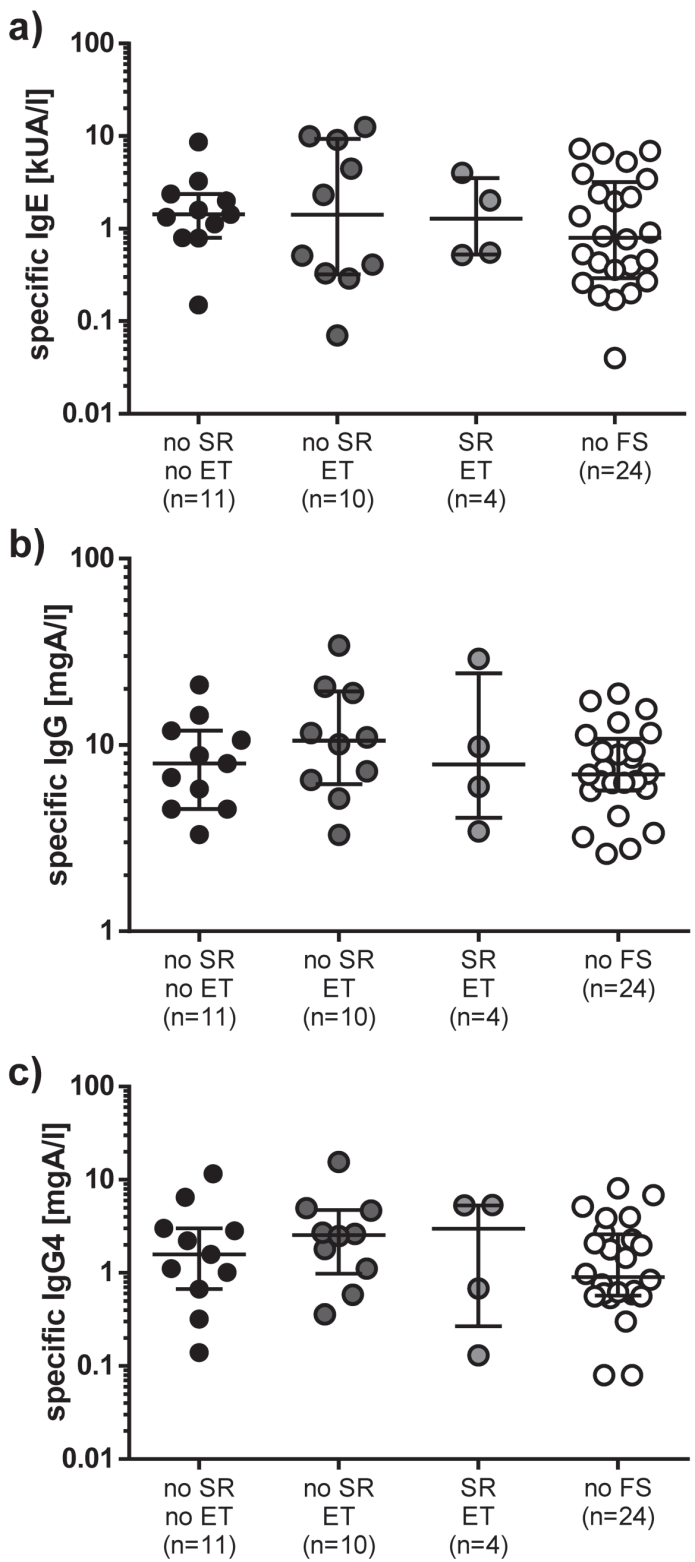
Concentrations of hymenoptera venom-specific (a) IgE, (b) IgG, and (c) IgG4 antibodies. ET = emergency treatment; FS = field sting; SR = systemic reaction.

Specific antibodies directed against the culprit insect causing the initial sting reaction could be analyzed in 49 patients who had stopped VIT 1 to 29 years ago. Median IgE serum concentrations did not differ between subjects who had or had not been stung again after finishing VIT, regardless of whether the patient was tolerant or had lost immune tolerance to HV (Figure 2a). In addition, HV-specific IgG and IgG4 antibodies were determined (Figure 2b, c) showing similar concentrations in individuals with SR to a re-sting and the other re-stung subjects. Likewise, no alterations were observed in the ratios of HV-specific IgE/IgG and IgE/IgG4 antibodies between the different groups (data not shown). 

### Psychosocial aspects 

The questionnaire encompassed three different topics, namely the long-term impact of VIT on 1) self-assurance, 2) daily life activities, and 3) personal environment (Table 2). Patients were given a total of 6 statements to which they documented their answers in a VAS. Analysis of the individual estimations was performed by comparing four groups of patients: 1) subjects with re-sting being HV-tolerant (no SR, no ET; n = 12), 2) subjects with re-sting who were not sure if they were HV-tolerant (no SR, but ET performed as a precaution; n = 11), 3) subjects with re-sting who had lost HV tolerance (SR, despite ET; n = 6), and 4) subjects with no re-sting (n = 25). 

**Long-term impact of VIT on self-assurance**

**Figure 3.**
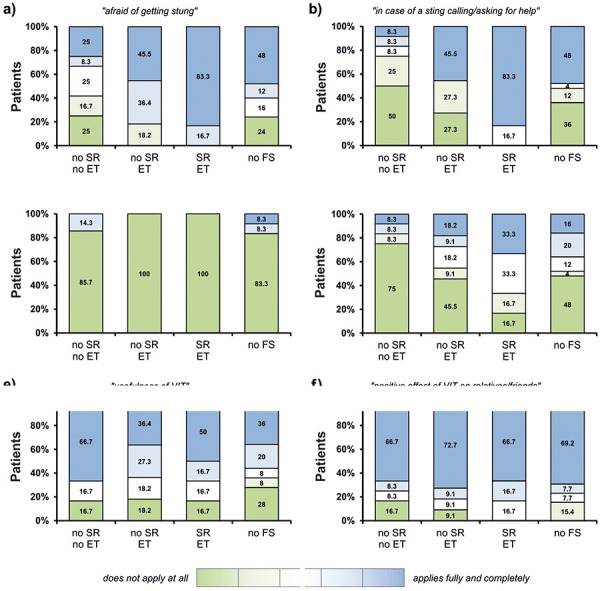
Results of the questionnaires on psychosocial aspects of VIT-treated patients with hymenoptera venom allergy. ET = emergency treatment; FS = field sting; SR = systemic reaction.

A tolerated hymenoptera sting had a strong impact on the self-assurance of HV-allergic patients. For instance, re-stung VIT-treated individuals not experiencing an SR and not taking any ET were markedly less afraid of getting stung again in comparison to all other subgroups (Figure 3a). On the other hand, patients who had lost their tolerance and developed an SR after a re-sting indicated the highest level of anxiety. Also, subjects who as a preventive measurement took ET after an HV sting and developed no anaphylactic reaction were still afraid of being stung again. Likewise, re-stung individuals who did not use ET and had no SR were least inclined to ask for help in case of future hymenoptera stings, whereas almost all of the patients who had suffered an SR expressed a great need of help (Figure 3b). 

**Long-term impact of VIT on daily life activities**

Most of the respondents did not feel restricted in their professional activities, most likely because there were no outdoor workers among them (Figure 3c). Yet, many of the interviewed subjects complained about restrictions in their leisure activities. However, while individuals with anaphylaxis after re-sting felt strongly impaired in their recreational activities, patients who were stung but did not develop an SR and did not need ET were by far the least severely affected, experiencing almost no restrictions (Figure 3d). Interestingly, also in the groups of patients unaware or doubtful of whether they really tolerate a hymenoptera sting, because they either prophylactically applied their emergency drugs or were not stung again, many subjects still considered themselves constrained during their leisure times. 

**Long-term impact of VIT on personal environment**

In line with the former results, re-stung subjects without SR and no ET were most confident about the usefulness of VIT (Figure 3e). Nevertheless, even patients who had lost their tolerance and experienced an SR after a re-sting as well as those who were not sure if they really were protected mostly valued VIT as beneficial. In terms of the perception of relatives and friends, the individual outcome of a re-sting did not differently influence their feelings and assumptions regarding the risks potentially imposed on their (formerly) HV-allergic family member or friend. Here, largely all considered VIT as a meaningful therapy positively affecting their QoL in the view of the interviewed patients (Figure 3f). 

**Long-term impact of VIT on overall quality of life**

Assessing the VIT-treated patients by a generic QoL instrument, the WHOQOL-BREF [31], neither their overall QoL nor one of the four domains, i.e., physical health, psychological health, social relationships or environment, were impaired compared to the general German population, indicating that their answers to the questionnaire were not affected by generally disturbed emotional believes or behaviors. 

## Discussion 

Approximately 40 years ago, Hunt et al. [2] convincingly showed that VIT is highly efficient in inducing tolerance in patients with HV allergy, resulting in protection against bee or wasp stings in 94.44% of treated subjects compared to 58.33% of placebo and 63.64% of whole-body extract-treated patients. Subsequent investigations have confirmed the efficiency of VIT, which has become the standard of care treatment in patients developing SR to hymenoptera stings [1]. However, despite the high success of VIT, allergen tolerance may not persist for a prolonged time. Thus, VIT-treated patients have to be aware that they might experience a relapse of their allergy, especially when stung again repeatedly [22, 24]. 

There have been several surveys executed since 1985 studying long-term persistence of tolerance to HV in VIT-treated patients (Table 3). While the majority has been performed in the U.S., with the remaining investigations conducted in different European countries, we here present for the first time since 1985 data from a German study cohort of adult individuals who had finished VIT 1 – 29 years prior. Follow-up in the previous studies lasted up to 20 years (range 1 month – 20 years). However, while 12 surveys covered a period of more than 5 years, just 7 extended the time frame of post-VIT observation beyond 10 years. Like for the post-VIT time, there was a great variability in the duration of VIT, comprising 1 month to 17 years (Table 3). While our cohort inclusion required as a minimum the recommended period of at least 3 years [1, 8], 8 of the previous surveys encompassed patients with a shorter VIT duration [9, 10, 12, 13, 15, 16, 19, 21]. Moreover, 5 of them encompassed individuals who had been treated for even only a few months, thus profoundly questioning the significance in reliably determining the long-term effect of VIT in these studies [10, 12, 15, 19, 21]. 

We assessed the status of HV allergy under real-life conditions by questioning tolerated FS, as it has been the case in two thirds (14/21) of the former studies, whereas in the other third this was done by performing SC. In one study, patients were evaluated either by FS or SC [24]. More than half of our patients (53.7%) experienced at least 1 re-sting after VIT had been terminated. This number corresponds to the upper range of the FS frequency in the other surveys (mean 42.25% of re-stung patients, range 20.8 – 92.2%) and is comparable to at least one (55.2% [25]) of the two investigations comprising similar long post-VIT observation periods of up to 20 years (Table 3). Approximately 20% of the re-stung patients from our study developed an SR and thus had lost HV tolerance. Considering the mean percentages of the other surveys (FS 10%, range 0 – 28.5%; SC 4.4%, range 0 – 19.7%), the number seems rather high but still equivalent (FS 19.2% [22]; SC 19.7% [18]) or even lower (FS 22%, 26.8%, 28.5% [10, 12, 19]) than found in a few other investigations. Differences may be due to the variable duration of both VIT and post-VIT time frames in the various surveys. In addition, the low frequency of only 3% relapse in a follow-up period of up to 20 years in one study may be mainly due to the fact that only children were investigated [9], who are known to show a favorable outcome in regard of re-sting SR [33, 34]. This is also the reason why we did not include further surveys only investigating HV-allergic children in our analysis. 

Notably, in the 9 studies following post-VIT patients for only 5 years, 7 evaluations showed re-sting SR rates of below 10% (3 of them 0%). Thus, the long-term protective effect of VIT might have been overestimated due to the circumstances that 1) patients may not be re-stung for a longer time period because of (initially) carefully exercising sting-avoidance measurements and 2) tolerance may vanish at later time points. In this regard, several of the previous surveys showed that quite a few patients re-experienced an anaphylactic reaction to HV after receiving at least one previous, at this occasion still tolerated bee or wasp sting, pointing to a boost of their HV allergy. 

It has been argued that declined allergen-specific IgE serum antibody titers may serve as an immune parameter linked to clinical allergen tolerance in HV-allergic subjects treated with VIT [13, 28, 35]. Comparing the different groups of our study cohort surveyed 1 – 29 years after stopping VIT in regard to reaction patterns to re-stings, no difference was found between subjects who were still protected or had lost HV-allergen tolerance. Studies following patients with either HV or pollen allergy after finishing immunotherapy have shown persistent levels of allergen-specific serum IgE antibodies despite tolerance induction [28, 36, 37], most likely due to the endurance of long-living antibody-secreting plasma cells, also called memory plasma cells [38]. Thus, allergen tolerance appears to be independent of allergen-specific IgE levels, rendering this parameter unsuitable for evaluation of long-term protection in VIT-treated subjects. We also analyzed HV-specific IgG and IgG4 antibodies in our patients, which are induced by allergen immunotherapy and are assumed to be protective by blocking allergen fixation by IgE antibodies [39]. However, the small number of surveyed patients with confirmed long-lasting HV tolerance experiencing no re-sting SR does not allow a conclusive statement about the significance of this measurement in evaluating long-term protection. Recently, we have shown that although the capacity of these IgG antibodies in preventing IgE binding to HV (in this case of the major wasp allergen Ves v 5) is still strongly enhanced in patients off VIT – some of whom were also part of the cohort investigated here – it is less than found during active immunotherapy [40]. However, while the IgG-dependent allergen blocking activity seems to be a more conclusive parameter correlated with clinical allergen tolerance than the IgG serum titer [41], further studies are needed to better estimate the value of this biomarker in assessing the long-term course of VIT-treated subjects after being off treatment. 

HV allergy has a substantial impact on QoL [42]. This is not surprising, since on the one hand, an insect sting might result in a severe, potentially life-threatening SR, and on the other hand, the encounter with the insect is not predictable and thus difficult to avoid. Consequently, the development of HV tolerance through VIT is associated with a considerable gain in QoL [3]. However, this seems to be substantially dependent on the experience of a tolerated sting [4, 5]. On the contrary, patients who have been treated with VIT and thus are highly likely to be protected when receiving a re-sting (with 80 – 95% being HV-tolerant [1]), but not having their tolerance confirmed by an SC are substantially restricted in their daily life due to the continuously perceived potential prevalence of their allergy [43]. Our survey, which is the first investigating personal behaviors and emotions connected to QoL issues in VIT-treated individuals over the course of many years after finishing VIT, confirms these findings. We here demonstrate that a tolerated re-sting helps further diminishing still prevailing and disturbing emotional distress aroused by thoughts of or a real encounter with hymenoptera species. Notably, not only patients suffering from a relapse of their allergy but also VIT-treated subjects who were not stung again and are thus potentially doubtful of their (lasting) HV tolerance seemed to be more affected than individuals experiencing a re-sting but still being protected. 

There are some limitations inherent to this study as it was a retrospective analysis encompassing a rather small number of patients and not including a negative, non-VIT-treated control population, thus not allowing statistically verifiable and conclusive general statements. Admittedly, a prospective setting continuously following HV-allergic patients after stopping VIT on a, for example, yearly basis would result in more informative data about the long-term course of HV allergy, especially since patients experiencing a re-sting are more likely to recall the exact circumstances and outcome. However, extraordinary high numbers of patients would be needed to retain a high quantity of them for such long periods of time. Furthermore, for ethical reasons, it is not possible to include a non-treated control cohort. 

In summary, this survey demonstrates that 1) a substantial percentage of VIT-treated patients show a long-term loss of HV tolerance, 2) measurements of HV-specific antibodies do not allow an estimation of the clinical status of continued tolerance, and 3) VIT-treated individuals are affected differently in their daily life by emotional distress depending on whether they experienced a re-sting and whether or not they developed an SR. Our findings of an increased re-sting frequency over time (53.7% in a follow-up period of up to 29 years), and at least one fifth of these (6/29) showing loss of tolerance, together with the data from previous reports, firmly strengthen the recommendation for long-term VIT of HV-allergic patients exhibiting risk factors for developing severe SR, like suffering from cardiovascular disease, asthma, clonal mast cell disorders, severe prior SR, increased age, or/and showing increased probability of being stung again due to bee-keeping, outdoor leisure activities, or professional hymenoptera exposure (e.g., working as a gardener, farmer, forest or construction worker, fruit or pastry seller [1, 8, 44, 45]). This might also hold true for HV-allergic subjects experiencing psychological or emotional distress when close to hymenoptera, which could have profound impact on behavioral patterns and personal feelings, as revealed by the answers to our questionnaire and by other similar investigations. In addition, it is advisable to routinely follow-up on HV-allergic patients, both those having stopped and those continuing VIT for extended time frames (e.g., every 2 years) to monitor the course of their allergy, give professional advice, and take proper action in terms of a relapse of their HV allergy [46]. 

## Essential sentence 

Long-term post-VIT analysis reveals that a considerable number of patients may experience loss of tolerance when stung again, but a tolerated re-sting after cessation of treatment is associated with substantially improved quality of life. 

## Funding 

No funding. 

## Conflict of interest 

J. Pickert has received lecture fees from ALK-Abelló, Novartis, and Sanofi Genzyme; W. Pfützner has received grants from Biomay AG, ALK-Abelló, and has consultant arrangements with ALK-Abelló and Thermo Fisher; J. Adelmeyer and C. Möbs declare that they have no relevant conflict of interest. 

**Table 1.** Patients with hymenoptera venom allergy.

Age (years)	59	(22 – 79)
Sex (female/male)	28/26	
Total IgE (kU/l)	38.7	(5.2 – 1,708)
VIT with	(n)	(%)
Wasp venom	40	74.1
Bee venom	10	18.5
Both	4	7.4
Systemic reaction (SR)^a^		
Grade I	3	5.56
Grade II	14	25.93
Grade III	30	55.56
Grade IV	1	1.85
N/A^b^	6	11.11
Field sting after VIT	29	53.7
No SR + no emergency treatment	12	22.2
No SR + emergency treatment	11	20.4
SR + emergency treatment	6	11.1

^a^According to Ring and Messmer; ^b^not applicable due to insufficient details for accurate grading.

**Table 2.** Questionnaire on psychosocial aspects.

Impact of VIT	Statements
… on self-assurance	I am very afraid of getting stung.
	In case of a sting I am calling/asking for help immediately.
… on daily life activities	I feel restricted during professional activities due to my allergy.
	I feel restricted during leisure activities due to my allergy.
… on personal environment	I am convinced of the usefulness of VIT.
	VIT positively affected the quality of life of my relatives and friends.

**Table 3.** Long-term surveys on patients with Hymenoptera venom allergy after finishing VIT.

Survey [reference]	Patients (n)	VIT duration	Post-VIT observation period	Patients with re-sting (% off all patients)	Patients with SR to re-sting (% of re-sting patients)
Reisman 1985 [10]	88	1 m – 6.5 y	1 m – 6 y	41 [FS] (46.5)	11 (26.8)
Urbanek 1985 [11]	31	n.s.	1 – 3 y	31 [SC] (100)	0 (0)
Golden 1986 [12]	82	2 – 44 m	9 – 79 m	28 [FS] (34.1)	6 (22)
Randolph 1986 [13]	57	1 – 8 y	up to 5 y	25 [FS] (43.8)	2 (8)
Golden 1989 [14]	30	5 – 8 y	1 y	29 [FS] (92.2)	0 (0)
Reisman 1989 [15]	194	6 m – 5 y	up to 11 y	79 [FS] (40.7)	8 (10.1)
Keating 1991 [16]	51	2 – 10 y	1 – 5 y	51 [SC] (100)	2 (3.9)
Haugaard 1991 [17]	25	3 – 7 y	1 – 3 y	25 [SC] (100)	0 (0)
Müller 1991 [18]	86	3 – 10 y	1 y	86 [SC] (100)	17 (19.7)
Reisman 1993 [19]	113	< 1 – > 5 y	up to 12 y	35 [FS] (30.9)	10 (28.5)
Golden 1996 [20]	74	≥ 5 y	5 y	74 [SC] (100)	7 (9.5)
v. Halteren 1997 [21]	75	< 1 – 10 y	3 y	75 [SC] (100)	6 (8)
Golden 1998 [22]	125	5 – 12 y	1 – 7 y	26 [FS] (20.8)	5 (19.2)
Lerch 1998 [23]	358	3 – 12 y	3 – 7 y	200 [FS] (55.8)	25 (12.5)
Golden 2000 [24]	194	5 – 17 y	1 – 13 y	81 [FS,SC] (46.2)	8 (9.1)
Golden* 2004 [9]	163	≤ 3 – ≥ 5 y	1 – 20 y	64 [FS] (39)	2 (3)
Hafner 2008 [25]	181	> 3 y	3 – 20 y	100 [FS] (55.2)	8 (8)
Erzen 2012 [26]	23	4 – 6.5 y	1 – 21 m	23 [SC] (100)	1 (4.4)
Ertoy Karagol* 2014 [27]	22	5 y	4 – 11 y	6 [FS] (27.2)	1 (16.6)
Pravettoni 2015 [28]	159	5 y	1 – 10 y	56 [FS] (35.2)	0 (0)
Albanesi 2018 [29]	23	50 – 73 m	27 – 199 m	14 [FS] (60.8)	3 (10)
This survey 2020	54	3 – 11 y	1 – 29 y	29 [FS] (53.7)	6 (20.7)

FS = field stings; m = months; n.s. = not specified; SC = sting challenges; SR = systemic reaction; y = years; * = survey of children
